# Dementia knowledge assessment scale (DKAS): confirmatory factor analysis and comparative subscale scores among an international cohort

**DOI:** 10.1186/s12877-017-0552-y

**Published:** 2017-07-31

**Authors:** Michael J. Annear, Chris Toye, Kate-Ellen J. Elliott, Frances McInerney, Claire Eccleston, Andrew Robinson

**Affiliations:** 1 Wicking Dementia Research and Education Centre, University of Tasmania, Hobart, TAS Australia; 2 School of Nursing, Midwifery and Paramedicine, Curtin University, Bentley, WA Australia; 3 School of Health Sciences, University of Tasmania, Hobart, TAS Australia

**Keywords:** Dementia, Knowledge, DKAS, Confirmatory factor analysis, Scale psychometrics

## Abstract

**Background:**

Dementia is a life-limiting condition that is increasing in global prevalence in line with population ageing. In this context, it is necessary to accurately measure dementia knowledge across a spectrum of health professional and lay populations with the aim of informing targeted educational interventions and improving literacy, care, and support.

Building on prior exploratory analysis, which informed the development of the preliminarily valid and reliable version of the Dementia Knowledge Assessment Scale (DKAS), a Confirmatory Factor Analysis (CFA) was performed to affirm construct validity and proposed subscales to further increase the measure’s utility for academics and educators.

**Methods:**

A large, de novo sample of 3649 volunteer respondents to a dementia-related online course was recruited to evaluate the performance of the DKAS and its proposed subscales. Respondents represented diverse cohorts, including health professionals, students, and members of the general public. Analyses included CFA (using structural equation modelling), measures of internal consistency (α), and non-parametric tests of subscale correlation (Spearman Correlation) and score differences between cohorts (Kruskal-Wallis one-way analysis of variance).

**Results:**

Findings of the CFA supported a 25-item, four-factor model for the DKAS with two items removed due to poor performance and one item moved between factors. The resultant model exhibited good reliability (α = .85; *ω*_*h*_ = .87; overall scale), with acceptable subscale internal consistency (α ≥ .65; subscales). Subscales showed acceptable correlation without any indication of redundancy. Finally, total and DKAS subscale scores showed good discrimination between cohorts of respondents who would be anticipated to hold different levels of knowledge on the basis of education or experience related to dementia.

**Conclusion:**

The DKAS has been confirmed as a reliable and valid measure of dementia knowledge for diverse populations that is capable of elucidating knowledge characteristics across four coherent domains: 1) Causes and Characteristics, 2) Communication and Behaviour, 3) Care Considerations, and 4) Risks and Health Promotion. Importantly, the four confirmed subscales clearly distinguish between groups who might be expected to hold differing levels of knowledge about dementia, allowing for a fine-grained level of detail to be established when evaluating baseline understanding or knowledge change associated with educational intervention.

## Background

The World Health Organisation recommends heightened dementia awareness and education among those who provide care and treatment in response to increasing global prevalence of this syndrome [1]. Target populations for education include health service workers [2], aged care staff [3], family caregivers [4], general practitioners (GPs) [5], and students from health-related disciplines [6]. Members of the general public also form an important learner cohort as the global rise in dementia will undoubtedly lead to increasing community contact with people who have dementia – necessitating dementia literacy [7]. Furthermore, increasingly, national government plans are being developed to facilitate inclusion of, and equity for, people living with dementia [8], meaning that policy makers and other professional groups, such as community housing developers and customer service representatives, will require understanding in this area.

In order to identify baseline understanding of dementia and the effects of educational interventions among such cohorts, it is necessary to develop valid and reliable measures. Such measures should not only include items addressing the biomedical aspects of the syndrome (pathology, causes, risk factors, and symptoms), but also address the psychosocial issues of care and communication – reflecting a holistic, biopsychosocial approach [9]. A biopsychosocial approach refers to a manner of viewing the progression of functional limitation in terms of all its dimensions (through effects on body, personhood, and social interaction) and is seen as a more effective understanding of chronic disease and its management [10]. One of the prevailing limitations of current dementia knowledge measures is their focus on a single domain of measurement and primacy of items addressing the biological elements of dementia. What is required is to ensure that the multi-faceted nature of dementia and dementia care can be adequately demonstrated in a measure with items and subscales that address other aspects of the condition, including care needs.

The development of dementia knowledge assessment tools draws on Bandura’s Social Cognitive Theory (SCT) [11]. In concordance with the health education literature [12, 13], we contend that learning experiences and knowledge acquisition concerning dementia care and treatment are a foundation of self-efficacy (belief in one’s ability to accomplish a task and/or provide care or treatment), which in turn underpins best-evidence behaviour towards a person with dementia. This assertion is supported by systematic review of evidence in chronic health management for older adults, which has found that higher levels of clinical knowledge resulting from workplace education portend better care outcomes mediated through changes in care and treatment behaviour [14]. Therefore, a reliable and valid Dementia Knowledge Assessment Scale (DKAS) helps to establish baseline knowledge and knowledge change parameters to support the development of educational resources and potentially influence improvements in care and treatment provision as well as supportive interactions with family and community members.

Prior to the development of the DKAS, measures of dementia knowledge had been tested and developed with relatively small (e.g. *N* < 500) and narrowly defined populations (e.g. undergraduate health students), lacked generalizability, focused mainly on biomedical domains or particular types or stages of dementia (e.g. Alzheimer’s disease), had ceiling effects among educated respondents, simplistic response categories, and item ambiguity [4, 15]. Beyond issues of dementia scale development and testing, definitive construct validity has seldom been confirmed and indicative subscales have not been verified. Confirmatory factor analysis (CFA) is part of the family of techniques used in structural equation modelling for the purposes of psychometric evaluation of measures, construct validation, and subscale confirmation [16]. Importantly, CFA allows for the testing and confirmation of a hypothesized model developed from earlier exploratory analyses [16, 17] (including principal components or exploratory factor analysis). In this case, it was appropriate to employ CFA to test previous assumptions (hypothesised factorial validity and indicative subscales) identified in a principal components analysis (PCA). CFA has seldom been employed in the verification of dementia knowledge scales, although it has previously been used to develop measures of depression among older adults [18, 19] and carer burden [20].

The 27-item DKAS was recently identified as a reliable and preliminarily valid measure of dementia knowledge among a diverse cohort of international and Australian respondents [17]. Factorial validity was established for the DKAS using PCA and four indicative subscales were proposed [17], which provide a balance between the measurement of biological and psychosocial domains. The creation of the DKAS drew on established scale development procedures [6, 21] and was informed by an international Delphi study of dementia experts to inform content selection [22]. Initial construction and piloting of the DKAS is described in detail elsewhere [17]. This measure has utility for researchers and educators who seek to delineate understanding of dementia among different cohorts or evaluate the efficacy of educational interventions. Further analysis of the 27-item DKAS with a large international cohort (including the country of study origin, Australia) has identified that the scale is more sensitive than other international measures, including the commonly used Alzheimer’s Disease Knowledge Scale (ADKS) [23].

This research sought to undertake a CFA with DKAS response data from a large international cohort, confirm the validity of four hypothesized subscales that resulted from a previously published PCA, determine potential differences in knowledge across diverse cohorts, and finalise scale refinement to establish its usefulness in evaluations of dementia knowledge and the efficacy of educational programs.

## Methods

### Sample, setting, and data collection

A large, de novo sample of international respondents was recruited from the third iteration (October 2014 to January 2015) of the *Understanding Dementia Massive Open Online Course* (UD MOOC) (October 2014 – January 2015) [24, 25]. Health professionals, students, family members of people with dementia, and the public were invited to participate at the beginning of the course after receiving information about the study. Participants self-selected into the cohort of DKAS responders. They were informed that scale completion counted as consent for de-identified and aggregated data to be used in analysis and reporting. A University Human Research Ethics Committee reviewed and approved this study (Ref no. H0013532).

### Measure

The DKAS [17, 23] comprises statements about the syndrome that are factually correct or incorrect, which were developed on the basis of a literature review and international Delphi study with dementia experts [22]. Respondents answer on a modified Likert scale with five response options: false, probably false, probably true, true, don’t know. Preliminary PCA identified four hypothesised components/subscales within the measure that have been defined as Causes and Characteristics (dementia pathology and terminal course), Communication and Behaviour (how a person with dementia engages with the world), Care Considerations (dementia symptoms relevant to the provision of care), and Risks and Health Promotion (risk factors and conditions that are associated with or mistaken for dementia) [23].

### Data analysis

All analyses were developed using SPSS [26]. Initial steps in the analysis included an evaluation of central tendency, missing data, and the potential impacts of outlier values. Data were non-normally distributed and exhibited a negative skew, which is common in social survey data [27]. Potential outlier effects were examined by comparing the 5% trimmed mean with the mean for the total sample. Evidence to support the construct validity of the DKAS was evaluated with the AMOS 20.0 package for SPSS [26] to conduct the CFA (using structural equation modelling). The CFA developed in this research was based on hypothesised subscales identified in a previous PCA with pilot data. There is some debate in the international literature concerning the use of PCA as a preliminary step in the development of a CFA, although the process aligns with published procedures for the development of other gerontological measures [18, 28, 29]. In this instance, a preliminary PCA was used principally as an item-reduction technique where no a-priori theorised data structure was applied and to identify a latent structure within the measure that could then by further confirmed via CFA.

CFA was performed using summary knowledge scores from the Dementia Knowledge Assessment Scale (DKAS) attained from a large sample of participants in an international dementia MOOC. It should be noted that CFA is robust to variations in respondent total scores as it focuses solely on the relationships between scale items and latent factors (potential subscales) based on a-priori hypotheses to confirm whether (or not) items truly define the construct that they are intended to define [30–32]. Additionally, with very large samples (*N* > 1000) distribution is also considered relatively inconsequential when using *asymptotically distribution free* (ADF) estimation, which avoids the assumptions of multivariate normality, and has been shown to produce factor loadings that are as accurate as estimation techniques for normally distributed data (i.e. maximum likelihood) under a range of conditions [33].

Following CFA and subsequent final refinement of the DKAS, measures of internal consistency (Cronbach’s alpha) for the overall measure and subscales were provided to assess reliability. Spearman Rho correlations were calculated to assess the level of correlation between subscale scores and as a measure of potential redundancy and duplication. Finally, total and subscale scores for different cohorts were considered in relation to the tool’s validity. The Kruskal-Wallis test was employed as a non-parametric assessment of differences among cohorts of DKAS completers for total score and subscale scores.

## Results

### Data characteristics and demographic information

A volunteer sample of 3649 respondents completed the DKAS at the outset of their participation in the online UD MOOC (2014/15). A total sample of 11,241 MOOC participants were invited to complete the DKAS and associated demographic questions, indicating a 32% response rate. Missing data among the respondents were not evident due to requirements for participants to complete all DKAS items before submitting the online survey form. Reasons for non-response were not provided as completion of the DKAS was entirely voluntary and administration was conducted in a virtual setting. Respondents from 97 countries were represented in the cohort of DKAS respondents. DKAS data were non-normally distributed and displayed a negative skew with no kurtosis evident. Examination of the 5% trimmed mean (38.78) revealed that it was not substantively different from the true mean (38.39), indicating negligible influence from high and low outlier values in relation to the total score. See Table 1 for demographic information.

**Table 1:** Respondent demographic information

Sample characteristics	MOOC international sample (n = 3649)
Mean age	47 years (*SD* = 13.03)
Age range	76 (14–90 years)
Male respondents	302 (8.3%)
Female respondents	3347 (91.7%)
Number of countries represented in DKAS responses	97
Main DKAS respondent countries	
Australia	1746 (48%)
United Kingdom	590 (16%)
New Zealand	239 (7%)
Canada	183 (5%)
United States	149 (4%)
Philippines	125 (3%)
Ireland	69 (2%)
India	61 (2%)
South Africa	35 (1%)
Germany	27 (1%)
Occupational groups	
General practitioner	26 (0.7%) Male (26.9%) Female (73.1%)
Nurse	918 (25.2%) Male (6.6%) Female (93.4%)
Health student	173 (4.7%) Male (13.9%) Female (86.1%)
Family carer	115 (3.2%) Male (3.5%) Female (96.5%)
Professional care worker	912 (25%) Male (7.6%) Female (92.4%)
Other health care worker	649 (17.8%) Male (5.9%) Female (94.1%)
General population	856 (23.5%) Male (14.4%) Female (85.6%)
Prior dementia education experiences	891 (24.4%)
Family member with dementia	1368 (37.5%)

### Confirmatory factory analysis

The 27-item, four-factor hypothesised model was examined using CFA based upon the preliminary findings of the PCA reported elsewhere [17]. The 27-item, four-factor model exhibited good model fit (GFI = .967; RMSEA = .042), however, correlation between the *Communication and Behaviour* and *Risks and Health Promotion* factors was high (.815), there was a perception of remaining redundancy among two items (with a degree of relatedness noted and correlation at the upper bound of acceptability for scale construction), and some item loadings resisted logical factor interpretations. The chi-square model summary was statistically significant for all variations of the model (*p* < .001), although this was affected by the very large sample size and was not a reliable measure of model fit in this case [34].

Two items were removed due to redundancy and/or poor fit within the confirmed model and one item was moved to a different factor to improve interpretation. Removing item 5 (most forms of dementia reduce the length of a person’s life) improved GFI (.973) and RMSEA (.040) and reduced the correlation between factors two (Communication and Behaviour) and four (Risks and Health Promotion) (.812). Removal of item 20 (people with dementia are unlikely to experience depression) further improved GFI (.974), maintained an acceptable RMSEA (.040), and reduced the estimated factor correlation between factor two (Communication and Behaviour) and factor four (Risks and Health Promotion) to a more acceptable value of .775. In order to further improve the face validity and factor interpretability, item 13 (people experiencing dementia do not generally have problems making decisions) was moved from factor one (Causes and Characteristics) to factor three (Care Considerations) – underlining the role that individual decision-making capability potentially plays in requirements for care as dementia progresses. Following this move, GFI, RMSEA, and correlations between factors remained unchanged, and factors one and three exhibited a clearer explanatory profile.

**Fig. 1:**
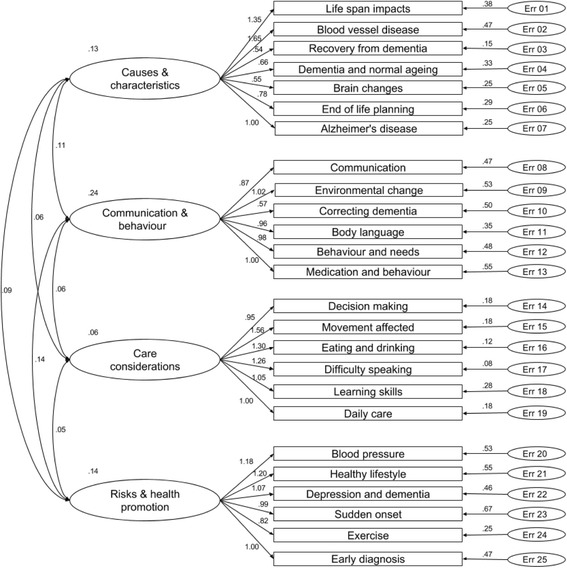
Confirmation of a four-factor, 25-item model for the DKAS

The resultant 25-item DKAS exhibited a high level of model fit (GFI = .974; RMSEA = .040), with four clearly discernible factors that were defined as, a) Causes and Characteristics (7 items), b) Communication and Behaviour (6 items), c) Care Considerations (6 items), and d) Risk Factors and Health Promotion (6 items) – largely confirming the hypothesised model, with some minor changes. Estimated inter-correlation between latent factors was acceptable, with all below the acceptability criterion of .80 [32]. The accepted CFA model is presented below in Fig. 1 (see Appendix for a full list of items and factor loadings).

### Internal consistency and subscale coherence

The Cronbach’s alpha and McDonald’s omega values for the overall scale indicated an acceptable level of internal consistency (α = .85; *ω*_*h*_ = .87), with no indication of redundancy. Cronbach alpha scores for each of the subscales ranged from .65 to .76 – surpassing or approaching acceptability criterion of >.70 and consistent with other validated scales reported in the health literature [35].These results indicate that there is an underlying association between each of the subscales within the 25 item DKAS, but that correlations are no so high as to indicate redundancy or thematic duplication across the four domains (Table 2).

**Table 2:** Cohort total and subscale pre-education scores for the 25-item DKAS

Cohort	Summative mean score (SD) / 50	Subscale mean scores (SD)			
		Causes & characteristics / 14	Communication & behaviours / 12	Care considerations / 12	Risks & health promotion / 12
Alpha (α)	α = .85	α = .69	α = .68	α = .76	α = .65
General practitioners (*n* = 26)	36.88 (9.78)	11.50 (3.10)	7.46 (3.42)	9.69 (2.84)	8.23 (3.31)
Nurses (*n* = 918)	37.89 (7.63)	11.52 (2.66)	8.28 (3.01)	10.62 (2.02)	7.47 (2.82)
Health students (*n* = 173)	34.48 (8.30)	10.73 (3.00)	7.17 (2.95)	10.00 (2.67)	6.57 (3.02)
Professional carers (*n* = 912)	34.53 (8.41)	10.73 (3.05)	7.48 (3.00)	10.26 (2.35)	6.06 (2.86)
Family carers (*n* = 115)	34.46 (6.75)	10.97 (2.74)	6.65 (2.84)	10.62 (1.81)	6.22 (2.59)
Other healthcare worker (*n* = 649)	37.08 (7.98)	11.54 (2.71)	8.07 (3.07)	10.38 (2.16)	7.09 (2.80)
General population (*n* = 856)	32.52 (9.05)	10.52 (3.19)	6.35 (3.22)	9.70 (2.65)	5.94 (2.86)

### Differences between groups

**Fig. 2:**
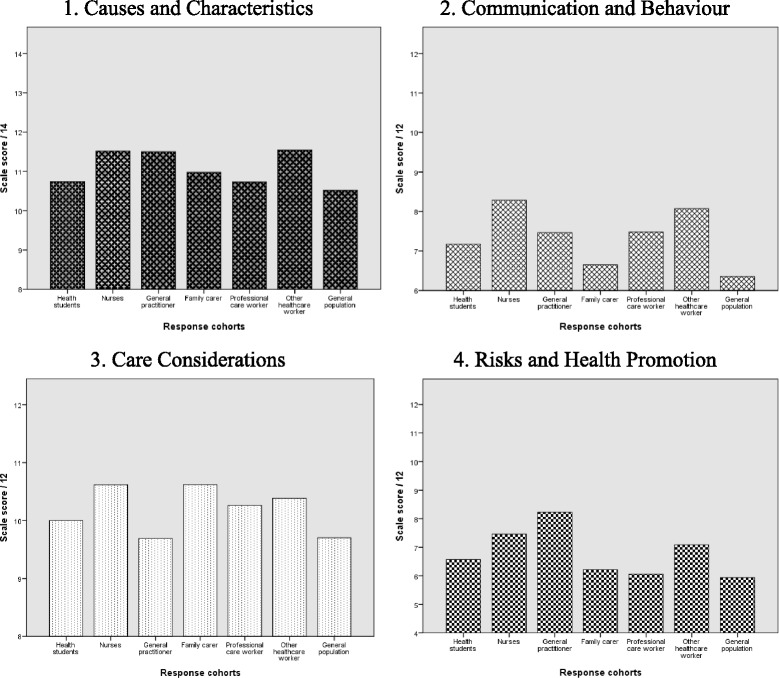
Subscale scores delineated by DKAS respondent cohort

The Kruskal-Walis Test revealed a statistically significant difference in DKAS scores (25-item) across 7 occupational groups, χ^2^ (6, *n* = 3623) = 206.39, *p* < .001. Highest scores were reported by qualified nurses and health care professionals. Comparatively lower scores were reported by health students, care workers, and family carers. Lowest scores were observed among a cohort from the general population. Across all four of the subscales within the DKAS, significant differences between occupational cohorts who could be expected to hold different levels of knowledge were also observed. A Kruskal Wallis test was also performed to examine whether significant differences in knowledge of dementia could be observed among different occupational cohorts. For each of the subscales, there were significant differences recorded among seven occupational cohorts: 1) Causes and Characteristics, χ^2^ (6, *n* = 3623) = 74.88, *p* < .001; 2) Communication and Behaviours, χ^2^ (6, *n* = 3623) = 199.54, *p* < .001; 3) Care Considerations, χ^2^ (6, *n* = 3623) = 65.03, *p* < .001; 4) Risks and Health Promotion, χ^2^ (6, *n* = 3623) = 185.56, *p* < .001. In Fig. 2, relative knowledge differences are shown between different response cohorts.

## Discussion

The DKAS has been identified as a reliable and preliminarily valid measure of dementia knowledge, which performs well when compared to existing, but relatively limited, measures. However, previous research has identified the need for CFA concerning the DKAS to verify construct validity (factorability), further refine the scale, and finalise four hypothesised subscales [17, 23]. CFA with dementia knowledge scales remains relatively uncommon in the international literature [15]. To the best of our knowledge, the DKAS is the only major measure of dementia knowledge to be subjected to this level of analysis using a diverse, international sample. Previously published PCA results identified a hypothesised four-component structure within the 27-item DKAS [17]. The construct validity of this model required verification with a large, de novo sample from among participants in an international dementia MOOC. Consistent with established best-practice analysis [30], CFA verified the construct validity of a 25-item iteration of the DKAS and supported refined iterations of the four indicative subscales that were initially observed in prior exploratory analysis. Some changes were made to further refine the DKAS as a result of the CFA procedures.

Two items were removed during the CFA analysis on the basis of statistical analysis to improve correlations between subscales, enhance factor interpretability, and achieve acceptable CFA model statistics. The conceptual domains of the scale were also considered during this process. Removal of item 5 (most forms of dementia reduce the length of a person’s life) was regarded as appropriate from a conceptual perspective considering that item 8 also addresses the life-limiting nature of dementia. However, removing item 20 (people with dementia are unlikely to experience depression [false]) was more contentious as the only other item addressing depression (item 11) referred to depressive symptoms being mistaken for manifestations of dementia rather than to prevalence. There is evidence that symptoms of depression in people living with dementia often go unrecognised in primary care, meaning that under-treatment becomes more likely [36]. Indeed, the prevalence of depression in people with dementia may be as high as 68% in some cases [37]. Including an item measuring the understanding of the higher prevalence of depressive symptoms was considered desirable. However, on balance, retaining this item was not considered the best option because this would undermine the statistical integrity of the CFA and, therefore, the validity of the four subscales. Movement of one item (item 13) between factors was consistent with the domains of the scale and improved factor interpretation without changing CFA model statistics. Thus, the definitive version of the English-language version of the DKAS is considered to be composed of 25 items and four subscales.

The findings of the present analysis suggest that the DKAS may now be used to effectively generate overall knowledge scores, subscale scores, and item-level metrics when administered with nurses, carers and family members of people with dementia, allied health workers, students in health-related disciplines, and members of the general adult population. More work is arguably required with a larger population of GPs due to the low number of participants in the present cohort. The four verified subscales within the 25-item DKAS mirror the thematic areas of the components identified during an earlier PCA with the 27-item pilot version – with minor refinements arising from the CFA procedures. Subscales include the following: a) Causes and Characteristics (7 items scored out of 14), b) Communication and Behaviour (6 items scored out of 12), c) Care Considerations (6 items scored out of 12), and d) Risk Factors and Health Promotion (6 items scored out of 12). Subscales are significantly correlated with each other above the benchmark level of .30 [38], although correlations are not high enough to suggest redundancy or duplication.

The overall and subscale scores can also measure significant differences between cohorts of test takers who could be expected to hold different levels of knowledge about the condition – affirming the construct validity of the 25-item DKAS. Of note, nurse respondents scored relatively highly on all subscales, GPs (from a comparatively small sample) scored relatively poorly on the Care Considerations and Communications and Behaviour subscales, family carers scored relatively highly on the Care Considerations scale and relatively poorly on the other scales, the general adult population recorded the lowest scores across all subscales. Previous studies of dementia knowledge have observed that understanding is associated with higher levels of health education [4, 39]. However, the utility of the revised DKAS measure rests in the capacity to distinguish expertise in different knowledge domains, including biomedical and care-related considerations. For example, high levels of nursing and family carer respondent knowledge with regards to care considerations relative to other cohorts, including a small population of GPs, reveals that understanding is not only delineated by previous education level, but also by experience and regular interaction with people who live with dementia. 

Importantly, and as alluded to in the introduction, the DKAS subscales provide measurement capability that moves beyond the biological and pathological bases of dementia. The scale also considers psychosocial aspects of the syndrome, including information about how a person with dementia engages with the world, symptoms that are relevant to the configuration and provision of care, and information about risk factors and conditions that are associated with or mistaken for dementia. Other measures of dementia knowledge focus predominantly on the biomedical aspects of the condition [15], so the verification of four diverse subscales within the DKAS provide a timely addition to the literature as dementia prevalence and consequent care requirements increase globally. In a systematic review of the dementia knowledge studies, Spector and colleagues [15] asserted that, “there is a need for a robust, contemporary measure which incorporates ‘biopsychosocial’ and ‘person centred’ models of care”. The refined DKAS fulfils this need enabling a more holistic understanding of dementia literacy and supporting the development of targeted educational interventions.

The results of this study were established with a large convenience sample. The cohorts of individuals who completed the DKAS had all sought out online dementia education and, therefore, potentially had a higher level of pre-existing understanding about the syndrome or greater motivation to learn, and possibly higher levels of education, than could be expected from a purely random sample. Notions of respondents’ pre-existing understanding were further reinforced as many respondents reported that they had a family member with dementia (37.5%) and/or experiences of previous dementia education (24.4%). In spite of this potential limitation, significant differences were still observed between respondent cohorts, indicating that the 25-item DKAS has excellent discriminative capability even among individuals who potentially have higher baseline knowledge. One minor concern in this study was that some participants may have partially completed part of the online course before submitting DKAS responses. However, this was not evident in the response distribution or outlier values (i.e. the 5% trimmed mean was consistent with the true mean and there was no evidence of bimodal distribution), and there is no reason to believe that greater numbers of any particular respondent cohort were involved in this late submission. Therefore, any influence on scores attained can be assumed to apply across all cohorts, meaning that comparisons among cohorts remain valid. Further work could be conducted with randomly sampled community cohorts with very low or varying baseline knowledge of dementia. Additional testing may also reinforce the reliability of factors with Cronbach’s alpha values lower than 0.7. While α values between 0.65 and 0.69 are considered minimally acceptable [40, 41], more work may be required with large, random samples to definitively assess their reliability. Additionally, the population of male respondents in this study was low, but this is consistent with the literature concerning aged and dementia care, where females make up the overwhelming majority of institutional and home care. Among the cohorts of individuals who completed the DKAS survey, the highest proportions of females were found among nurses, professional carers, family carers, and other healthcare professionals. International research has identified dementia care is a heavily gendered issue and found that, “across all regions and settings, females bear the brunt of the incidence of dementia and the responsibility for caring for people with dementia” (p. 44) [42]. Although females dominate in aged and dementia care, more work is potentially required with male health professionals and carers to understand the role of gender in dementia knowledge. Further work is also required to test the revised DKAS with those who may be expected to hold higher levels of knowledge about dementia, including GPs (acknowledging that GP engagement with people with dementia can be highly episodic), neurologists, psychogeriatricians, and neuropsychologists. GPs and other highly qualified health professionals can be among the most challenging cohorts to recruit into research due to significant demands on their time and competing priorities. Because a comparatively small cohort of GPs completed the DKAS (*n* = 26) as part of UD MOOC participation, work is currently underway to establish that reliability and validity of the DKAS and its subscales with a larger sample of GP registrars and medical educators. In order to establish the DKAS as a global measure of dementia knowledge, testing in other languages is also recommended. A Japanese-language version of the DKAS (the DKAS-J) has been successfully piloted [43] and validation in other languages is also indicated. Super-aging societies [44] in the Asia-Pacific region, including Japan, Taiwan, Singapore, and China may be appropriate locations for ongoing testing of the measure due to the anticipated substantial increase in dementia prevalence in such locations.

The confirmation of four stable subscales within the 25-item DKAS increases the utility of the measure. It can now be used to create a total summative score, item scores, or subscale scores in the areas of disease characteristics, care considerations, communication, and behaviour. The capacity to measure dementia knowledge among diverse populations, across varied domains of understanding, and at fine-grained levels of detail, will have utility to accurately assess understanding and help to develop targeted educational interventions. Such a measure is likely to be particularly useful to translational health service researchers, educationalists within the health sciences, public health policy planners, and health advocacy organisations, To the best of our knowledge, this is the first time that a dementia knowledge scale has been created with a full confirmatory factor analysis and an international sample to identify underlying subscales. The DKAS is likely to be particularly useful for identifying specific problems with dementia literacy and thereby informing more effective targeting of interventions designed to address knowledge deficits. In our view, this greatly adds to the utility of the DKAS as a tool and enables researchers to target specific problems with dementia literacy in specific groups and design and implement interventions to address these problems. This is important because the evidence suggests significant knowledge deficits within health and aged care professionals and family carers [4], as well as undergraduate nursing students [45] who will take a key role in providing dementia care into the future. This situation must be addressed if people with dementia and their carers are to receive evidence-based, high-quality care and support. The existence of a robust DKAS with subscales addressing diverse facets of dementia will strategically support this effort. Congruent with established theory in health behaviour and education [11–13], it is anticipated that accurate assessment of dementia knowledge deficits and needs for enhanced dementia literacy will ultimately lead to improved care for people with dementia as the self-efficacy and treatment/care behaviours of health providers and family members is improved by resultant educational interventions.

## Conclusion

The 25-item, four subscale DKAS is a reliable and valid measure of dementia knowledge for health professionals, trainees, and members of the general public that is capable of elucidating knowledge characteristics across four coherent domains: 1) Causes and Characteristics, 2) Communication and Behaviour, 3) Care Considerations, and 4) Risks and Health Promotion. Importantly, the four confirmed subscales clearly distinguish between groups who might be expected to hold differing levels of knowledge about dementia, allowing for a fine-grained level of detail to be established when evaluating baseline understanding or knowledge change following educational intervention. Future work is indicated with GPs, large random samples, and in other languages to continue to build on the development of the measure.

## Appendix

**Table 3:** The 25-item DKAS, confirmed domains, and standardised factor loadings

Item #	Statement content	Domains / factor names	Standardised loadings
1	Most forms of dementia do not generally shorten a person’s life [*False*]	Causes and characteristics	.616
2	Blood vessel disease (vascular dementia) is the most common form of dementia [*False*]	Causes and characteristics	.651
3	People can recover from the most common forms of dementia [*False*]	Causes and characteristics	.447
4	Dementia is a normal part of the ageing process [*False*]	Causes and characteristics	.382
5	Dementia does not result from physical changes in the brain [*False*]	Causes and characteristics	.369
6	Planning for end of life care is generally not necessary following a diagnosis of dementia [*False*]	Causes and characteristics	.463
7	Alzheimer’s disease is the most common form of dementia [*True*]	Causes and characteristics	.578
8	It is impossible to communicate with a person who has advanced dementia [*False*]	Communication and behaviour	.529
9	A person experiencing advanced dementia will not generally respond to changes in their physical environment [*False*]	Communication and behaviour	.572
10	It is important to correct a person with dementia when they are confused [*False*]	Communication and behaviour	.369
11	People experiencing advanced dementia often communicate through body language [*True*]	Communication and behaviour	.627
12	Uncharacteristic behaviours in a person experiencing dementia are generally a response to unmet needs [*True*]	Communication and behaviour	.574
13	Medications are the most effective way of treating behavioural symptoms of dementia [*False*]	Communication and behaviour	.554
14	People experiencing dementia do not generally have problems making decisions [*False*]	Care considerations	.477
15	Movement is generally affected in the later stages of dementia [*True*]	Care considerations	.664
16	Difficulty eating and drinking generally occurs in the later stages of dementia [*True*]	Care considerations	.668
17	People with advanced dementia may have difficulty speaking [*True*]	Care considerations	.729
18	People experiencing dementia often have difficulty learning new skills [*True*]	Care considerations	.429
19	Daily care for a person with advanced dementia is effective when it focuses on providing comfort [*True*]	Care considerations	.498
20	Having high blood pressure increases a person’s risk of developing dementia [*True*]	Risks and health promotion	.520
21	Maintaining a healthy lifestyle does not reduce the risk of developing the most common forms of dementia [*False*]	Risks and health promotion	.521
22	Symptoms of depression can be mistaken for symptoms of dementia [*True*]	Risks and health promotion	.510
23	The sudden onset of cognitive problems is characteristic of common forms of dementia [*False*]	Risks and health promotion	.412
24	Exercise is generally beneficial for people experiencing dementia [*True*]	Risks and health promotion	.526
25	Early diagnosis of dementia does not generally improve quality of life for people experiencing the condition [*False*]	Risks and health promotion	.482

## Abbreviations

ADF: Asymptotically Distribution Free
CFA: Confirmatory Factor Analysis
DKAS: Dementia Knowledge Assessment Scale
GP: General Practitioner
PCA: Principal Components Analysis
UD MOOC: Understanding Dementia Massive Open Online Course
